# Sangivamycin is highly effective against SARS-CoV-2 in vitro and has favorable drug properties

**DOI:** 10.1172/jci.insight.153165

**Published:** 2022-01-11

**Authors:** Ryan P. Bennett, Elena N. Postnikova, Brett P. Eaton, Yingyun Cai, Shuiqing Yu, Charles O. Smith, Janie Liang, Huanying Zhou, Gregory A. Kocher, Michael J. Murphy, Harold C. Smith, Jens H. Kuhn

**Affiliations:** 1 OyaGen, Inc., Rochester, New York, USA.; 2 Integrated Research Facility at Fort Detrick, National Institute of Allergy and Infectious Diseases, National Institutes of Health (NIH), Frederick, Maryland, USA.; 3 Center for Musculoskeletal Research, University of Rochester School of Medicine & Dentistry, Rochester, New York, USA.

**Keywords:** COVID-19, Therapeutics, Drug therapy

## Abstract

Sangivamycin is a nucleoside analog that is well tolerated by humans and broadly active against phylogenetically distinct viruses, including arenaviruses, filoviruses, and orthopoxviruses. Here, we show that sangivamycin is a potent antiviral against multiple variants of replicative severe acute respiratory syndrome coronavirus 2 (SARS-CoV-2) with half-maximal inhibitory concentration in the nanomolar range in several cell types. Sangivamycin suppressed SARS-CoV-2 replication with greater efficacy than remdesivir (another broad-spectrum nucleoside analog). When we investigated sangivamycin’s potential for clinical administration, pharmacokinetic; absorption, distribution, metabolism, and excretion (ADME); and toxicity properties were found to be favorable. When tested in combination with remdesivir, efficacy was additive rather than competitive against SARS-CoV-2. The proven safety in humans, long half-life, potent antiviral activity (compared to remdesivir), and combinatorial potential suggest that sangivamycin is likely to be efficacious alone or in combination therapy to suppress viremia in patients. Sangivamycin may also have the ability to help combat drug-resistant or vaccine-escaping SARS-CoV-2 variants since it is antivirally active against several tested variants. Our results support the pursuit of sangivamycin for further preclinical and clinical development as a potential coronavirus disease 2019 therapeutic.

## Graphical abstract

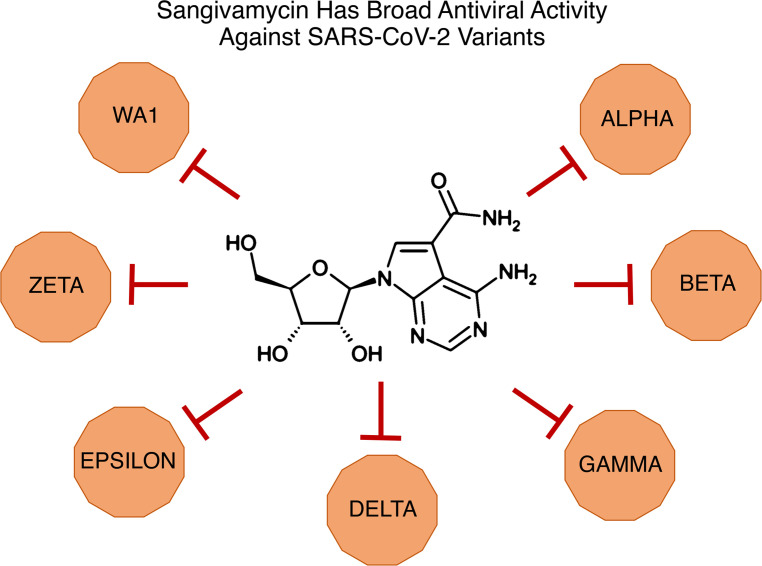

## Introduction

Severe acute respiratory syndrome coronavirus 2 (SARS-CoV-2), a sarbecovirus in the genus *Betacoronavirus* (Nidovirales: Coronaviridae), is the etiologic agent of coronavirus disease 19 (COVID-19) (1–3). COVID-19 was first documented in Wǔhàn, Húběi province, China, in late 2019 (4–6) and has since spread rapidly across the globe, leading WHO to declare a pandemic on March 11, 2020 (7). Typically, SARS-CoV-2 infection is asymptomatic/subclinical or presents as a mild upper respiratory disease (self-limiting fever, cough, nonspecific fatigue, and myalgia), but, in a substantial number of cases (typically elderly and/or comorbid), it involves the lower respiratory tract, leading to pneumonia that may progress to acute respiratory distress syndrome and death (8–14). Thus far, 241 million cases of SARS-CoV-2 have been reported worldwide, including close to 5 million attributable deaths (15). Several vaccines are now widely available, and numerous others are in development, for the prevention of COVID-19 (16). However, treatment options for COVID-19 cases remain mainly limited to supportive care, which has had to be aggressive in severe cases. Antiviral options approved for treatment by the US FDA include the small molecule remdesivir along with 2 anti–SARS-CoV-2 monoclonal antibody combinations (bamlanivimab/etesevimab and casirivimab/imdevimab) under emergency use authorization (17). Because large populations may not have the opportunity or access, may decline, or may not be sufficiently healthy to receive vaccinations, there is an urgent need for the development of new highly efficacious drugs to improve the prognosis and long-term outcomes of hospitalized patients with COVID-19 and to counter possible vaccine-escaping and/or drug-resistant SARS-CoV-2 variants.

We recently reported that the adenosine nucleoside analog sangivamycin, an unsuccessful anticancer drug candidate that nevertheless was proved to be safe in 88 humans tested in clinical trials by the US National Cancer Institute (NCI) in the 1960s (18), is a potent, dose-dependent inhibitor of arenaviruses (Lassa virus), filoviruses (Ebola virus and Marburg virus), and orthomyxoviruses (cowpox virus and vaccinia virus) with an IC_50_ in the nanomolar range in grivet (*Chlorocebus aethiops*) kidney epithelial Vero E6 and human hepatocellular carcinoma Huh-7 cells (19). Because of its broad-spectrum activity and its long half-life in tissues (20), we hypothesized that sangivamycin may be active against SARS-CoV-2. Using replicative SARS-CoV-2, we show that sangivamycin was indeed highly active against multiple variants (including Delta) of the virus with an IC_50_ in the nanomolar range in several cell types. Sangivamycin suppressed SARS-CoV-2 replication with greater potency than remdesivir and had an additive effect on virus infection rate when combined with remdesivir. We further demonstrate that sangivamycin’s properties are favorable for further preclinical and clinical development.

## Results

### Sangivamycin inhibits SARS-CoV-2 infection in multiple cell types.

We previously demonstrated low nanomolar efficacy of sangivamycin against distinct viruses, including Ebola virus and Marburg virus (Filoviridae), Lassa virus (Arenaviridae), and cowpox virus and vaccinia virus (Poxviridae), in grivet Vero E6 and human Huh-7 cells (19). Based on this broad spectrum, we hypothesized that sangivamycin would also inhibit SARS-CoV-2. Vero E6, human colorectal adenocarcinoma Caco-2, and human lung Calu-3 cells were pretreated with sangivamycin for 1 hour at various doses and then exposed to SARS-CoV-2 at MOIs optimized for maximum infectivity for each cell type. Cells were fixed and SARS-CoV-2 infection rate was quantified using high-content imaging following immunostaining with a primary antibody reactive with the SARS-CoV-2 nucleoprotein and a fluorescently labeled secondary antibody, with percentage infectivity measured as the number of SARS-CoV-2–positive cells relative to total cells measured by Hoechst 33342 nuclear staining (Supplemental Figure 1A; supplemental material available online with this article; https://doi.org/10.1172/jci.insight.153165DS1). As a baseline, cytotoxicity was measured with a CellTiter-Glo assay examining ATP levels to determine cell viability in mock-exposed cells grown in parallel to virus-exposed cells.

**Figure 1:**
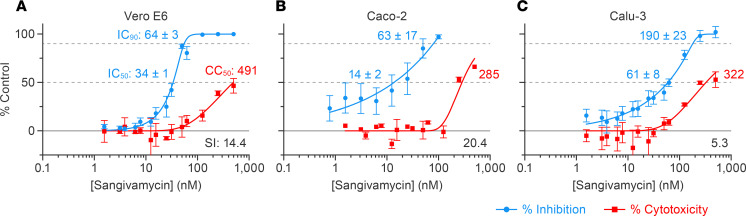
Sangivamycin inhibits SARS-CoV-2 replication in multiple cell types. (**A**–**C**) High-content imaging assays were performed to determine compound potency (blue line), and CellTiter-Glo assays were performed to determine cell viability (red line) of sangivamycin-pretreated cells exposed to SARS-CoV-2. Results are reported as percentage inhibition (blue values) and cytotoxicity (red values) relative to untreated controls. Error bars represent standard deviations (SDs) from tests run for each concentration in triplicate on 3 plates for Vero E6 and Calu-3 (*n* = 9) and sextuplet on 4 plates for Caco-2 (*n* = 24). IC_50_, half-maximal inhibitory concentration (lower dotted line); IC_90_, 90% inhibitory concentration (upper dotted line); CC_50_, half-maximal cytotoxic concentration (lower dotted line); SI (bottom right), selectivity index (CC_50_/IC_50_). ±, standard error of the mean (SEM) across plates (*n* = 3 for Vero E6 and Calu-3, *n* = 4 for Caco-2).

Vero E6 cells are commonly used in virus drug screens and hence were used for initial experiments. Sangivamycin’s antiviral activity in Vero E6 cells, including the IC_50_, 90% inhibitory concentration (IC_90_), half-maximal cytotoxic concentration (CC_50_), and selectivity index (SI = CC_50_/IC_50_), are shown in Table 1 (WA1) and graphed in Figure 1A. As expected, SARS-CoV-2 infection rate was high, even at a low MOI of 0.012 (77% ± 4% SEM SARS-CoV-2–positive cells in untreated control cells across 3 plates). Sangivamycin’s antiviral activity was equally effective at higher MOIs, ranging from 0.2 to 1.3, with an average IC_50_ of 52 ± 13 nM (SEM) (Supplemental Table 1). Of immediate interest was the finding that at MOIs of 0.2 and 0.4, GS-441524 (remdesivir’s parent nucleoside) had an IC_50_ of 1420 ± 20 nM (SEM), which was 27-fold higher than the IC_50_ of sangivamycin at the same MOIs (Supplemental Table 1).

In vivo, both lung and intestinal cells grow with directionality, depending on which surface is exposed to air in lungs or lumen in the gut. Therefore, Caco-2 and Calu-3 cells required assay optimization prior to compound testing to achieve optimal SARS-CoV-2 infection rate at the assay endpoint. Following optimization of assay conditions, we observed 25% ± 6% (SEM) SARS-CoV-2–positive cells in Caco-2 cells (optimized to MOI = 0.5, 96 hours incubation) and 76% ± 10% (SEM) SARS-CoV-2–positive cells in Calu-3 cells (optimized to MOI = 2, 72 hours incubation) (Supplemental Figure 1, B and C). Most importantly, sangivamycin proved very potent against SARS-CoV-2 in both cell types (Figure 1, B and C).

### Sangivamycin is more efficacious against SARS-CoV-2 than remdesivir in multiple cell types.

**Figure 2:**
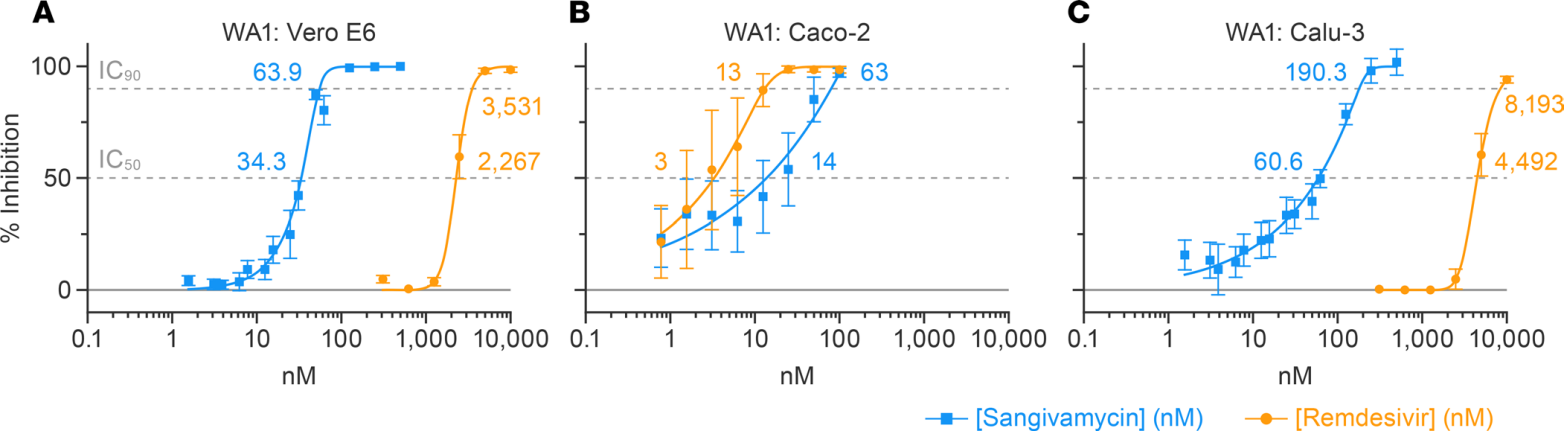
Sangivamycin is a more potent antiviral against SARS-CoV-2 than remdesivir in multiple cell types in vitro. (**A**–**C**) Sangivamycin’s antiviral activity (Figure 1) compared with remdesivir using identical SARS-CoV-2 MOIs and sampling time points. Results are reported as percentage inhibition relative to untreated controls (blue values for sangivamycin, yellow values for remdesivir). Error bars represent SDs for sangivamycin as described in Figure 1, and for remdesivir each concentration was run in triplicate on 3 plates for Vero E6 and Calu-3 (*n* = 9) and triplicate on 4 plates for Caco-2 (*n* = 12). IC_50_, half-maximal inhibitory concentration (lower dotted line); IC_90_, 90% inhibitory concentration (upper dotted line).

Remdesivir is currently the only small molecule approved by the FDA for treatment of COVID-19. We therefore compared the potencies of sangivamycin and remdesivir against SARS-CoV-2 in 3 cell types. In Vero E6 cells, sangivamycin’s IC_50_ and IC_90_ values were 66- and 55-fold lower than those of remdesivir (Figure 2A and Table 2). We measured a similar differential when we compared sangivamycin to remdesivir’s parent nucleoside, GS-441524 (Supplemental Table 1). In Caco-2 cells, which were not highly susceptible to SARS-CoV-2 infection (25%, compared with 77% in Vero E6 and 76% in Calu-3 cells), remdesivir robustly inhibited the virus, with IC_50_ and IC_90_ values in a similar low nanomolar range as sangivamycin (Figure 2B). In contrast, Calu-3 cell results mirrored those of Vero E6 in that sangivamycin’s IC_50_ and IC_90_ values were 74- and 43-fold lower than those of remdesivir (Figure 2C and Table 2). In fact, sangivamycin’s effective concentration was also lower than that of remdesivir against all SARS-CoV-2 variants tested in Vero E6 and Calu-3 cells (Table 2 and Supplemental Figure 2).

### Sangivamycin is efficacious against multiple variants of SARS-CoV-2.

**Figure 3:**
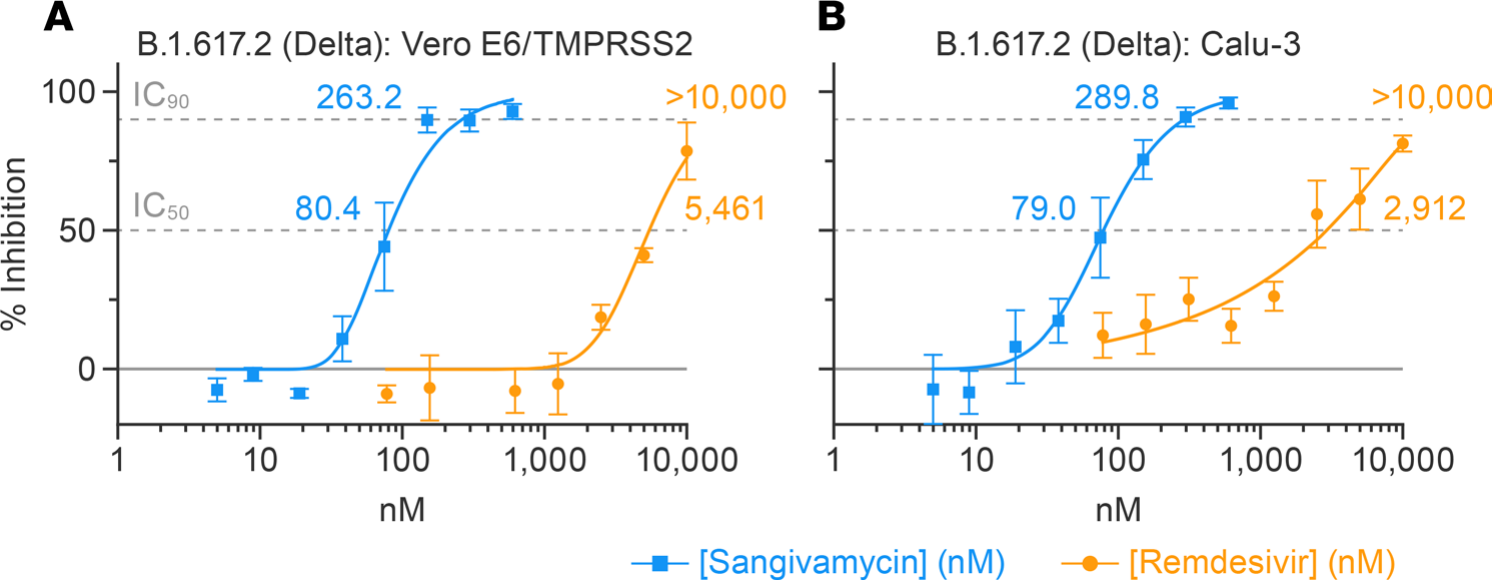
Sangivamycin is more potent against the Delta variant of SARS-CoV-2 than remdesivir. Sangivamycin’s antiviral activity compared with remdesivir using SARS-CoV-2 MOIs and sampling time points as detailed in Table 1 for SARS-CoV-2 variant B.1.617.2 (Delta) in Vero E6/TMPRSS2 (**A**) and Calu-3 (**B**) cells. Results are reported as percentage inhibition relative to untreated controls (blue values for sangivamycin, yellow values for remdesivir). Each dose was run in triplicate with error bars representing SDs. IC_50_, half-maximal inhibitory concentration (lower dotted line); IC_90_, 90% inhibitory concentration (upper dotted line).

Multiple variants of SARS-CoV-2 have spread globally since the COVID-19 outbreak began. Each new variant has the potential to evade vaccines and therapies. Therefore, we tested whether sangivamycin would maintain robust antiviral efficacy against multiple variants. Currently, the Delta variant has been the predominant variant in the United States since July 2021, according to the US Centers for Disease Control and Prevention (CDC). Unfortunately, Vero E6 cells were not susceptible to Delta variant infection. Therefore, we used Vero E6 cells expressing the transmembrane serine protease, TMPRSS2, which can be infected by SARS-CoV-2 (21). Vero E6/TMPRSS2 cells and Calu-3 cells were highly infected by the Delta variant, and sangivamycin had robust antiviral activity against the Delta variant, with IC_50_ values of 80 nM (Vero E6/TMPRSS2) and 79 nM (Calu-3) compared with remdesivir, which had much higher IC_50_ values of 5461 nM (Vero E6/TMPRSS2) and 2912 nM (Calu-3) (Table 2 and Figure 3, A and B). Six other viral variants were tested in both Vero E6 and Calu-3 cells. All variants were optimized for MOI and peak infection profile in each cell type. Sangivamycin was efficacious in the low nanomolar range against all variants (Table 1) in both cell types and continued to be several-fold more potent than remdesivir (Table 2 and Supplemental Figure 2, A–L).

### Combining sangivamycin and remdesivir results in an additive effect against SARS-CoV-2 in multiple cell types.

**Figure 4:**
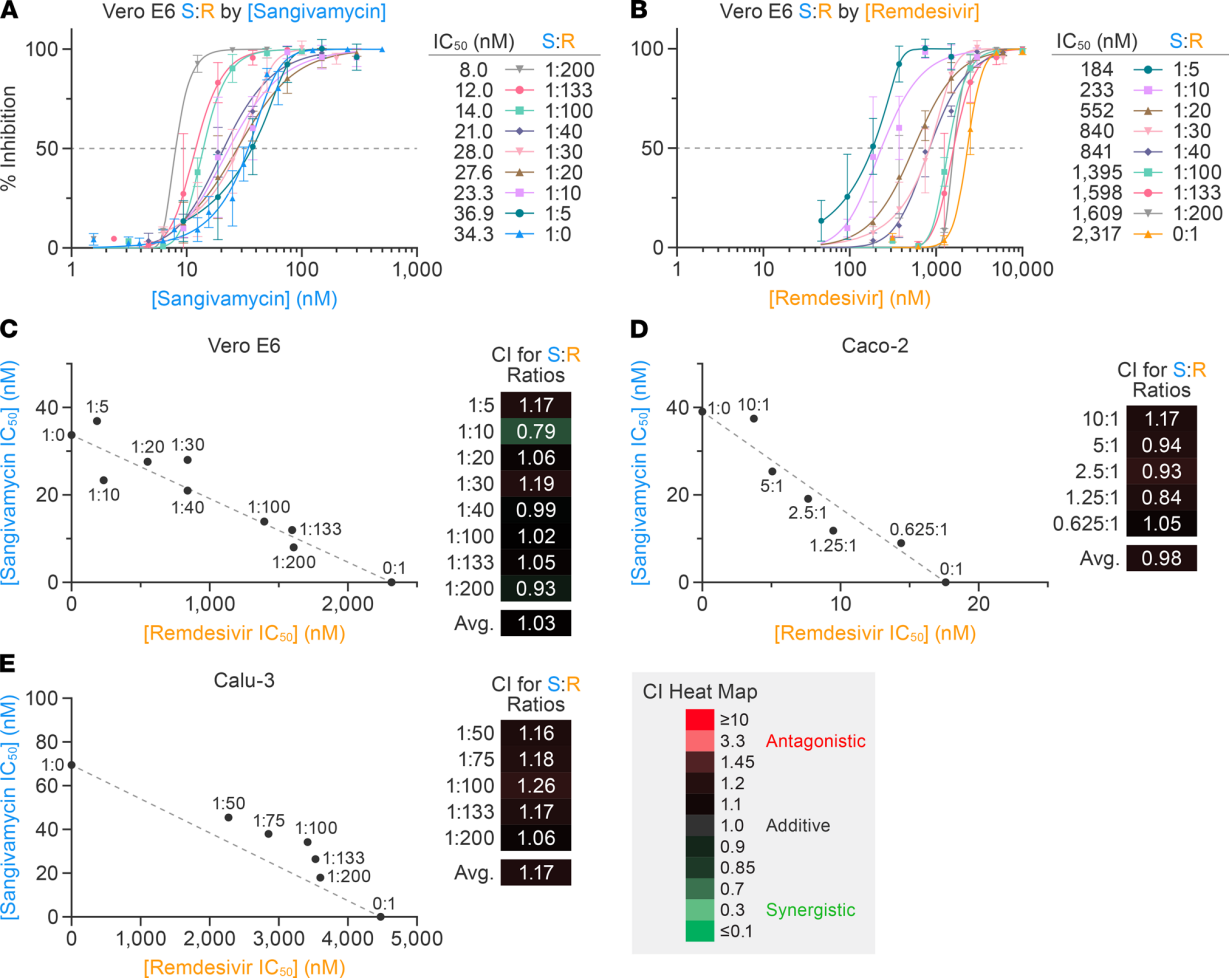
Combining sangivamycin and remdesivir results in an additive effect against SARS-CoV-2 in multiple cell types. Constant ratios of sangivamycin to remdesivir (S:R) were used to evaluate combination effect against SARS-CoV-2 infection in Vero E6 cells and plotted relative to (**A**) sangivamycin concentration and (**B**) remdesivir concentration. Each dose combination was run in triplicate with error bars representing standard deviations (SDs). (**C**) Effects of different combinations of sangivamycin-to-remdesivir ratios on viral infection rate, fit to the Loewe interaction model. Isobologram showing ratio pairs that resulted in 50% virus inhibition calculated from the curves in **A** and **B** plotted on the *y* axis (values from **A**) and *x* axis (values from **B**) relative to the additive (dotted) line drawn between the IC_50_ values for sangivamycin (S:R = 1:0) and remdesivir (S:R = 0:1) alone. The results of experiments similar to those shown in **A** and **B** performed on Caco-2 and Calu-3 cells are shown in Supplemental Figure 3. (**D** and **E**) Isobolograms as in **C** calculated based on results in Supplemental Figure 3. The CI heatmap legend indicates color coding for S:R antagonism (*red*), additive efficacy (*black*), or synergy (*green*) based on ref. 23. CI, combination index.

Remdesivir and sangivamycin are both nucleoside analogs that, when combined, have an additive effect in vitro in an Ebola virus minigenome cell-based assay that measures RNA-directed RNA polymerase (RdRp) activity (19). The Loewe additivity model is typically used as a reference model when the combined effect of 2 drugs is additive, and isobologram and combination index (CI) analyses are widely used to evaluate drug interactions. Therefore, we tested the combination of remdesivir and sangivamycin at several fixed ratios relative to their respective IC_50_ values to determine whether they act antagonistically, additively, or synergistically against SARS-CoV-2. Increasing concentrations of remdesivir relative to sangivamycin (Figure 4A) and increasing amounts of sangivamycin relative to remdesivir (Figure 4B) both lowered the IC_50_ values of each of the respective treatments. These values were plotted for each constant ratio of sangivamycin to remdesivir in an isobologram (22, 23). A straight line between the IC_50_ for sangivamycin alone (34 nM) on the *y* axis to the IC_50_ for remdesivir alone (2317 nM) on the *x* axis determined the additive line as assessed by the Loewe additivity model (Figure 4C). The results suggested that both compounds are acting on the same target in a noncompetitive manner.

The CI is a quantitative measurement for assessing drug efficacy in combination studies as defined by Chou et al., who originally proposed the CI metric and its utility in predicting drug combination outcomes (23). The CI values for each ratio of sangivamycin and remdesivir are listed next to isobolograms using a color-coded heatmap indicating relative synergy, additivity, and antagonism. The average CI for SARS-CoV-2–infected Vero E6 cells was 1.03, which is well within the additive range (Figure 4C). Similarly, isobolograms and CI values for Caco-2 (Figure 4D) and Calu-3 cells (Figure 4E) supported that a combination of sangivamycin and remdesivir resulted in additive effects, lowering the amount of each compound necessary to achieve an IC_50_ in both Caco-2 and Calu-3 cells (Supplemental Figure 3).

### Sangivamycin is not cytotoxic in vitro at antiviral doses.

Sangivamycin inhibits cellular kinases that are overexpressed in some cancer cells — most notably protein kinase C and histone H3 associated protein kinase — leading to apoptosis in pancreatic cancer cells, breast cancer MCF-AR cells, and primary effusion lymphoma cells. However, sangivamycin is well tolerated in normal pancreatic cells, Ramos cells, Burkitt lymphoma DG75 cells, and breast cancer MCF-WT cells, with cytostatic effects only becoming apparent at the high nanomolar to low micromolar dose range in MCF-WT cells (24–26). To further understand sangivamycin’s antiviral mechanism, we evaluated cell viability. Both the CellTiter-Glo luminescence-based assay (a measure of cellular ATP levels, Promega) and the CellTiter 96 AQueous One Solution cell proliferation (MTS) assay (a measure of the reduction of the tetrazolium salt by enzymes in metabolically active cells, Promega) were used to assess cell viability at 24 hours, 48 hours, and 72 hours in Vero E6 and Calu-3 cells. Both methods resulted in similar slow decreases in cell viability over time, but the decrease was more substantial in Vero E6 cells likely because Vero E6’s doubling rate is around 24 hours versus Calu-3, which double around 72 hours (Supplemental Figure 4) (27, 28). This growth rate–dependent effect is more consistent with cytostaticity in the high nanomolar range reported in the literature for normal cell types than the resulting cytotoxicity in kinase-overexpressing cancer cell types (24–26). However, it is notable that the antiviral activity is well below the cytostatic effect range, and the use of both Vero E6 and Calu-3 cells resulted in similar ranges for antiviral efficacy, suggesting that the antiviral mechanism is independent of the effects on cell growth.

Both CellTiter-Glo and the MTS assays measure overall cell viability compared with untreated control cells but are unable to determine whether the lower cell density is due to cytostatic or cytotoxic effects. In contrast, the CellTox Green Cytotoxicity Assay (Promega) determines cell viability based on membrane integrity as revealed by amount of a fluorescent DNA-binding dye capable of entering cells as a function of leaky membranes due to overtly cytotoxic outcomes (i.e., cell death). To confirm a cytostatic effect of sangivamycin, CellTiter-Glo and CellTox Green were used side by side using multiple cell types, including kidney (Vero E6 and HEK293T), liver (Huh-7, HepG2, and primary hepatocytes), intestine (Caco-2), and lung (Calu-3 and A549) cells that had been dosed with sangivamycin. The ratio of CellTox Green to CellTiter-Glo CC_50_ values can indicate cytotoxic versus cytostatic effects: a ratio > 1 indicates a cytostatic effect, and a ratio ≤ 1 indicates a cytotoxic effect. The average ratio for all cell lines tested with both assays (*n* = 6) was >12.2 ± 3.78, revealing a cytostatic effect in all cell types tested (Table 3 and Supplemental Figure 5). CellTiter-Glo evaluation resulted in an average CC_50_ of 617 ± 181 nM (SEM; *n* = 8) compared with a much higher average of more than 5000 nM determined by CellTox Green (Table 3). The average IC_50_ for all viral testing in all cell lines and MOIs was 47 ± 5 (*n* = 22, from Figure 1, Table 1, and Supplemental Table 1). Calculating the SI with the average CellTiter-Glo values gave a value of 13, whereas calculating SI with the average CellTox Green revealed a much higher SI (>106).

Other important measures of cell viability are mitochondrial integrity, function, cardiotoxicity, and genotoxicity, since other nucleoside analogs may vary in their effect on these endpoints (29, 30). Dyes commonly used to assess mitochondrial integrity are MitoTracker Green (MTG), a mitochondrial stain mostly localized to mitochondria based on hydrophobicity, and *N*-alkyl acridine orange (NAO), which binds to mitochondrial cardiolipin. Untreated Huh-7 cells and Huh-7 cells treated for 5 days with 100 nM sangivamycin were stained with MTG and NAO and imaged by fluorescence microscopy. Visual comparison of the fluorescent images suggested that sangivamycin did not significantly affect mitochondrial structural integrity (Table 4 and Supplemental Figure 6). To more quantitatively assess the impact of sangivamycin on mitochondrial integrity, we evaluated the effect of sangivamycin on mitochondrial metabolic function as well as cytoplasmic glycolytic function. The XF96e Seahorse instrument was used to quantify the oxygen consumption rate (OCR) over time in untreated control and sangivamycin-treated Huh-7 cells. Treatment with up to 5 μM of sangivamycin did not significantly change the OCR relative to the OCR measured in untreated control cells run in parallel. Standard metabolic endpoints quantified with or without sangivamycin included sequential treatment and washout of cell cultures with oligomycin (ATP synthase inhibitor), carbonyl cyanide 4-(trifluoromethoxy)phenylhydrazone (FCCP; a proton gradient uncoupler), and antimycin A + rotenone (complex I and III blockers) (Supplemental Figure 7). The data indicated that there was little to no significant acute inhibition of mitochondrial or glycolytic functions by sangivamycin (Table 4).

The KCNH2 (hERG) functions as the α subunit of a cardiac potassium channel. Its inhibition by drugs has been implicated in fatal ventricular tachyarrhythmia (31). As a predictor of cardiotoxicity and genotoxicity, we therefore evaluated KCNH2 (hERG) inhibition and genotoxicity in the AMES mutagenicity assay. Table 4 shows that 10 μM of sangivamycin had no effect on the KCNH2 (hERG) in cardiomyocytes and that there was no evidence of enhanced bacterial mutagenicity at up to 3.2 mM of sangivamycin.

### Sangivamycin does not induce phospholipidosis.

Certain cationic amphiphilic drugs cause phospholipidosis that results in an anti–SARS-CoV-2 effect in vitro that does not recapitulate in vivo (32). Although sangivamycin is not a cationic amphiphilic molecule and is not suspected to affect phospholipid storage, we tested whether sangivamycin could induce phospholipidosis. *N*-(7-nitrobenz-2-oxa-1,3-diazol-4-yl)-1,2-dihexadecanoyl-*sn*-glycero-3-phosphoethanolamine, triethylammonium salt (NBD-PE), is a phospholipid that fluoresces in protonic environments and is a reporter for phospholipidosis (32). Amiodarone served as a positive control for inducing phospholipidosis and induced NBD-PE fluorescence in treated A549 cells. In contrast, no significant NBD-PE fluorescence above the DMSO control baseline was evident when A549 cells were treated with up to 5 μM sangivamycin or the negative control compound, 10 μM melperone (Supplemental Figure 8).

### Sangivamycin has favorable drug properties.

In vitro absorption, distribution, metabolism, and excretion (ADME) studies were used to assess sangivamycin’s solubility, cytochrome P450 (CYP) inhibition, plasma protein binding, and liver microsome stability. Sangivamycin was soluble up to the high test concentration of 500 μM in phosphate-buffered saline (PBS) (Table 5). As expected, control CYP active compounds inhibited their respective CYP isoforms, but sangivamycin did not significantly affect CYP isoforms in the tested concentration range (1–100 μM), and any observed inhibition was insufficient to determine an IC_50_ (Table 5).

A decrease of test compound concentration over time during incubation with active microsomes indicates metabolization of the compound. A dose of 5 μM of sangivamycin was not metabolized by either human or mouse liver microsomes during 120 minutes of incubation (Table 5).

Binding to plasma proteins influences the plasma concentration of free drug reservoir and kinetics determining drug availability for distribution into tissues in the body. Only a low fraction of sangivamycin bound to plasma proteins (Table 5).

We next evaluated the 24-hour pharmacokinetics (PK) of sangivamycin in CD-1 laboratory mouse plasma. Sangivamycin’s plasma concentrations increased following i.p. injection of 60 mg/kg to a peak at 2 hours, then decreased over 6 or 8 hours, and remained virtually unchanged up to 24 hours after dosing. The C_max_ of the plasma was 97.3 ± 31.0 ng/mL (males) and 285 ± 35.9 ng/mL (females), observed at the maximum time of 2 hours. Graphing plasma concentration versus time produced an area under the curve to the last time point with values of 632 ± 66.6 h•ng/mL for males and 833 ± 53.9 h•ng/mL for females. The apparent volume of distribution was 369,000 mL/kg (males) and 167,000 mL/kg (females), and total clearance was 85,400 mL/h/kg (males) and 68,500 mL/h/kg (females) (Supplemental Figure 9A). Our data corroborate historical PK studies in mice with ^3^H-labeled sangivamycin performed at the NCI in 1974 (20). In these studies, free nucleoside and mono-, di-, and triphosphate forms of sangivamycin were detected in blood, urine, and multiple tissues (e.g., heart, liver, brain, spleen, and kidneys) following i.p. injection of 8 mg/kg. The studies established that sangivamycin was phosphorylated after transportation into cells. In liver, heart, and brain, the predominant form of sangivamycin was sangivamycin 5′-monophosphate (SMP). In these tissues, as well as in kidney and spleen, small amounts of RNA and DNA were found to contain drug-derived radioactivity that peaked after 24 hours and steadily decreased out to day 12, indicative of the di- and triphosphates being formed as well (20). Therefore, we determined the level of SMP in blood in our PK study using a published liquid chromatography–tandem mass spectrometry (LC-MS/MS) method with a lower limit of quantification of 1 ng/mL (33). Using this method, we detected SMP in the plasma of dosed mice but not control mice (Supplemental Figure 9B). The apparent levels of SMP increased in blood during the 24 hours of this study. There also was possible presence of a diphosphate metabolite of sangivamycin, but it was detected only in the plasma samples taken 24 hours after dosing.

## Discussion

At the time of this study, remdesivir is the only FDA-approved small molecule drug for COVID-19 treatment, and assessments of its clinical effectiveness through January 2021 continue to suggest that it increases the odds of clinical improvement (34). There is an urgent need for other immune system–independent treatment modalities. Molnupiravir is a candidate as a treatment of higher risk individuals that can result in 30% less risk for hospitalization and death based on an FDA advisory committee analysis of a phase III clinical trial with 1433 patients (35).

In our study, sangivamycin proved active against SARS-CoV-2 with IC_50_ values in the low nanomolar range (14–82 nM) against all viral variants and in all examined cell types (Figure 1 and Table 1). Importantly, sangivamycin’s activity was significantly lower than that of remdesivir in those experiments (Table 2, Figures 2 and 3, and Supplemental Figure 2). In addition, sangivamycin’s activity added to that of remdesivir in combinatorial experiments (Figure 4). Because combination therapies may be far more effective in suppressing viremia compared with single treatments and thereby also may hinder the emergence of drug-resistant virus variants, sangivamycin could be developed as a component of combinatorial drug products following efficacy vetting in an animal model.

The additive results indicate that sangivamycin does not antagonize the antiviral mechanism or metabolism of remdesivir, which requires metabolic processing to the triphosphate form to act as a delayed chain terminator for the SARS-CoV-2 RdRp (36). Sangivamycin also adds to the antiviral effect of remdesivir in the Ebola virus minigenome assay (our previous study, ref. 19). Those results suggested that sangivamycin is a nucleoside analog that may target RdRp function. Whether this effect is due to metabolic processing of sangivamycin to the triphosphate remains to be determined with ongoing in vitro mechanistic studies. Interestingly, sangivamycin also affected Ebola virus particle formation and release through interference with the Ebola virus matrix protein (VP40) in the absence of RdRp (19). This observation leaves open the possibility that sangivamycin may counteract SARS-CoV-2 in an RdRp-independent manner. Interestingly, there are nucleotide binding sites on nonstructural protein 12 (nsp12) within the SARS-CoV-2 RdRp holoenzyme and the auxiliary protein nsp13, which has helicase activity. Consequently, one may hypothesize that sangivamycin or its phosphorylated metabolites could bind to nucleotide binding sites in nsp 12 and 13 and affect their function. Moreover, sangivamycin incorporated in viral progeny RNAs could disrupt nsp13 helicase activity, which is crucial for viral replication and template switching during transcription of viral genes (37). We cannot rule out that sangivamycin, if incorporated in viral RNA, may affect RNA secondary structure, RNA stability, or RNA function in protein binding or translation.

The favorable ADME (Table 5) and PK, shown here and in the historical reports, indicate that sangivamycin may be superior to remdesivir because the metabolism of sangivamycin is slower and more uniform in maintaining phosphorylated forms in tissues. Remdesivir and its monophosphate are rapidly lost within 10 minutes in laboratory mouse liver, lung, and kidney microsomes (38), whereas 100% of sangivamycin remained after 120 minutes in our laboratory mouse and human liver microsome tests (Table 5). Furthermore, we showed that the level of sangivamycin remained high in plasma throughout the 24-hour study, and the increase in levels of SMP aligns with the previous NCI PK study (20). Hardesty et al. reported that the persistence of sangivamycin-derived radioactivity in tissues over a period of 12 days was a reflection on both (a) its conversion to SMP, which is trapped inside the cell; and (b) that sangivamycin is not susceptible to degradation by adenosine deaminase. The levels of sangivamycin and SMP were maintained at high levels in all tissues up to 6 days without a significant drop in levels with a half-life in blood of 50 hours (20). The main route of excretion appeared to be the kidneys; 40% of the drug-derived radioactivity was accounted for in the urine over a period of 12 days (20). In fact, the C_max_ of 97.3 and 285 ng/mL in our laboratory mouse PK study is equivalent to 314 nM and 922 nM sangivamycin, which is 3- to 9-fold above the average IC_90_ in the 3 cell lines tested (Figure 1). Notably, these were only plasma levels, but based on the Hardesty et al. report, cellular and tissue levels will be significantly higher than plasma levels (20). In contrast, remdesivir metabolism varies among cells and among tissues. In mice, as both the free nucleoside and monophosphate forms, remdesivir half-lives are only about 5 hours (38, 39). In fact, a recent PK analysis based on allometric scaling predicted that antiviral levels of remdesivir are not maintained in the current dosing regimen for patients with COVID-19 (40).

In the early 1960s, sangivamycin was evaluated in a phase I clinical trial by Pfizer in collaboration with the NCI as a potential cancer therapy. In several different animal models, 15 in vivo preclinical studies supported the safety results of the original clinical trial, including toxicology, histopathology, topical application of the intended clinical formulation, sphygmometry, and cardiotoxicity studies. Maximum tolerated doses (MTDs) were determined in mice, rats, dogs, and nonhuman primates. Preclinical work archived at the NCI revealed that sangivamycin was tested in grivets and was tolerated for 10 days at 1.6 mg/kg/d (total dose 16 mg/kg) and 28 days at 0.4 mg/kg/d (total dose 11.2 mg/kg) (41). In 40 human cancer patients, the compound proved inactive against the disease but was well tolerated with daily, thrice weekly, or weekly dosing (0.1–2.83 mg/kg total dose), and the MTD was not reached (18).

Extrapolating from in vitro antiviral testing (Figure 1 and Table 1), we anticipate sangivamycin doses necessary to achieve anti–SARS-CoV-2 IC_50_ and IC_90_ in human patients to be well within the safe dosing range based on these previous animal and human studies (18, 41). Indeed, we estimated the amount of sangivamycin in a 2-compartment model for a 70 kg adult (extracellular and cellular water volumes 42 L), indicating that the highest single dose safely tested in humans (0.3 mg/kg) would be 30-fold above the average in vitro IC_50_ from all cell types tested (47 nM or 0.01 mg/kg). Our experimental results support this safety profile (Tables 3 and 4), showing that sangivamycin would not display toxic effects at and above antiviral concentrations. Our data also suggest that the antiviral activity of sangivamycin is unlikely an artifact of phospholipidosis, mitochondrial toxicity, or cytotoxicity.

In summary, we suggest that sangivamycin has appropriate properties for treating SARS-CoV-2 infections based on data showing low nanomolar IC_50_ and IC_90_ antiviral activity against multiple cell types and viral variants, along with favorable PK and safety profiles. The extensive historical safety data in animals and human clinical trials, along with long tissue half-life, support the case for further development. Furthermore, the additive effect of sangivamycin with remdesivir suggests that treatment for COVID-19 with a combination of the 2 drugs could have therapeutic advantages, including lower doses of both drugs to achieve viral replication suppression and consequently reduced potential for side effects. Further investigational new drug–enabling preclinical studies are ongoing in preparation for regulatory approval for sangivamycin’s clinical development.

## Methods

### Cells.

Grivet (*Chlorocebus aethiops*) kidney epithelial Vero (American Type Culture Collection [ATCC]; CCL-81) and Vero E6 (BEI Resources; NR596), Vero E6/TMPRSS2 (JCRB Cell Bank, Japan; JCRB1819), human colorectal adenocarcinoma Caco-2 (ATCC; HTB-37), HEK293T (ATCC; CRL-3216), human hepatocarcinoma HepG2 (ATCC; #HB-8065), human hepatocarcinoma Huh-7 (a gift from NIH/National Institute of Allergy and Infectious Diseases [NIAID]/Rocky Mountain Laboratories, Hamilton, Montana, USA), human lung adenocarcinoma Calu-3 (ATCC; HTB-55), and human lung carcinoma A549 cells (a gift from University of Rochester Medical Center) were maintained at 37°C and 5% CO_2_ in DMEM (Life Technologies) containing 10% heat-inactivated FBS. Primary human hepatocytes from an anonymous 41-year-old woman with colorectal cancer metastasized to the liver were obtained from Liver Center Resources, Pittsburgh Liver Research Center, Pittsburgh, Pennsylvania, USA, and maintained at 37°C and 5% CO_2_ in Eagle’s modified essential medium (Gibco).

### Virus.

SARS-CoV-2 (Coronaviridae: *Sarbecovirus*) isolate SARS-CoV-2/human/USA-WA1/2020 (GenBank MN985325) was obtained from the CDC at passage 3 from sample collection. Virus was inoculated onto Vero cells and incubated for 72 hours in MEM (Gibco) containing 2% heat-inactivated FBS at 37°C and 5% CO_2_. The resulting virus stock (GenBank MW161259; internal reference IRF0394) was titrated by plaque assay as previously described using Vero E6 cells and a 2.5% Avicel overlay (FMC BioPolymer), followed by staining at 48 hours using 0.2% aqueous Gentian Violet (Ricca Chemicals) (42). The subsequent working stock (GenBank MT952134, internal reference IRF0399) was generated by infecting Vero cells with IRF0394 at an MOI of 0.01 for 48 hours. Media were collected after 3 freeze-thaw cycles. The stock was clarified via centrifugation at 7500*g* for 10 minutes. Both stocks tested negative for bacterial, endotoxic, and fungal contamination. Bacterial testing consisted of a 14-day incubation period in Tryptic Soy Broth (Corning) in a humidified incubator at 25°C and 37°C. Endotoxin and mycoplasma testing used an Endosafe cartridge system (Charles River Laboratories) and the Universal Mycoplasma Detection Kit (ATCC), respectively. SARS-CoV-2 variants were produced in the same manner as described above, and specific information about their lineage, WHO name, IRF lot number, source isolate, production cell line, and GenBank number are listed in Supplemental Table 2.

### Single-compound dose-response studies.

Sangivamycin nucleoside was obtained from Berry & Associates, Dexter, Michigan, USA. Remdesivir was obtained from Biosynth-Carbosynth, Itasca, Illinois, USA. GS-441524 was obtained from Target Molecule, Wellesley Hills, Massachusetts, USA. Black opaque (for cytotoxicity assays) or clear-bottom 96-well (for efficacy assays) Operetta plates (Greiner Bio-One) were seeded with 30,000 Vero E6 cells per well, 20,000 Caco-2 cells per well, or 50,000 Calu-3 cells per well 1 day prior to compound treatment. Compounds were first dissolved in DMSO (MilliporeSigma), and then stock solutions in media were diluted to 0.05% DMSO before adding to cells. For each assay, 6-point to 14-point dose-response with 2-fold step dilution was prepared. Each dose was evaluated in triplicate on 3 (Vero E6 and Calu-3 cells) or in sextuplicate for sangivamycin and triplicate for remdesivir on 4 separate plates (Caco-2 cells). For each plate, 4 and 10 μM chloroquine (MilliporeSigma) in duplicate served as the reference control for assay performance. The remaining wells on each plate were distributed among untreated and mock-infected negative controls (normalized to 0% infection rate) and untreated virus-exposed positive controls (normalized to 100% infection rate). Cells were pretreated with each dose of the compounds 1 hour prior to SARS-CoV-2 exposure. Exposures were performed at the following MOIs: 0.012, 0.2, 0.4, 0.6, and 1.3 (Vero E6 cells), 0.5 (Caco-2 cells), and 2 (Calu-3 cells). After 48 hours (Vero E6 cells), 96 hours (Caco-2 cells), and 72 hours (Calu-3 cells), cells were fixed with 10% neutral-buffered formalin (VWR), washed with PBS (Fisher Bioreagents), and blocked with PBS containing 3% w/v of BSA (MilliporeSigma). Cells were then stained with a SARS-CoV-2–cross-reactive primary rabbit monoclonal antibody targeting the severe acute respiratory syndrome coronavirus nucleoprotein (Sino Biological catalog 40143-R001) diluted 1:8000. Following PBS washes, cells were stained with fluorescently labeled goat anti–rabbit IgG secondary antibody (Thermo Fisher Scientific catalog A11037) and Hoechst 33342 (Thermo Fisher Scientific), both diluted at 1:2500. Fluorescence was measured using an Operetta High-Content Imaging System (PerkinElmer) with subsequent analysis using Harmony v4.9 software (PerkinElmer). Signal-to-noise ratios and Z′-factor scores were determined for all plates/wells for quality control purposes. IC_50_s, IC_90_s, CC_50_s, and SIs (CC_50_/IC_50_) were calculated with Prism v9.2.0 (GraphPad Software). SARS-CoV-2 variants were tested with the same method but in 384-well format and at the MOI and time points shown in Table 1.

### Combinatorial compound efficacy testing.

Optimal sangivamycin-to-remdesivir (S/R) constant ratios were established based on the single-compound IC_50_ ranges determined for each cell line (Vero E6, Caco-2, Calu-3). Two combination assays were performed with different compound dose ratios for Vero E6 cells. One experiment resulted in S/R constant dose ratios of 1:5, 1:10, 1:20, 1:30, and 1:40, with a sangivamycin concentration range of 1.5–300 nM and remdesivir concentrations of 46–6000 nM; the second experiment resulted in S/R constant ratios of 1:100, 1:133, and 1:200, with sangivamycin concentrations of 1.5–100 nM and remdesivir concentrations of 313–10,000 nM. Curves of 1:0 and 0:1 were derived from the single-compound tests. The constant ratios and concentrations evaluated for sangivamycin and remdesivir for Caco-2 cells were 10:1, 5:1, 2.5:1, 1.25:1, and 0.625:1, with sangivamycin concentrations of 3.13–100 nM and remdesivir concentrations of 0.625–20 nM, and ranges for Calu-3 cells were 1:50, 1:75, 1:100, 1:133, and 1:200, with sangivamycin concentrations of 1.5–100 nM and remdesivir concentrations of 313–10,000 nM. Constant S/R ratios were set up in triplicate for each concentration over 6-point dose-response curves with 2-fold compound dilutions evaluated as for each combination assay. CIs were calculated as CI = D_1_/(Dx)_1_ + D_2_/(Dx)_2_, where D_1_ and D_2_ are the respective combination doses of sangivamycin and remdesivir that yield 50% virus inhibition (IC_50_ values), and (Dx)_1_ and (Dx)_2_ are the respective IC_50_ values of sangivamycin and remdesivir alone (23).

### Cytotoxicity testing.

Sangivamycin cytotoxicity across the tested dose ranges was determined in parallel to antiviral efficacy using compound-treated mock-exposed cells (compound and media only) via the CellTiter-Glo Luminescent Cell Viability Assay (Promega) at 48 hours (Vero E6), 96 hours (Caco-2 cells), and 72 hours (Calu-3 cells) after treatment, according to the manufacturer’s instructions in triplicate. CellTiter-Glo testing for A549, HEK293T, human hepatocytes, Huh-7, and HepG2 cells was performed at 72 hours after treatment in triplicate. The MTS [CellTiter 96 AQueous One Solution Cell Proliferation 3-(4,5-dimethylthiazol-2-yl)-5-(3-carboxymethoxyphenyl)-2-(4-sulfophenyl)-2H-tetrazolium, inner salt] Assay (Promega) was performed according to the manufacturer’s instructions at 24, 48, and 72 hours after treatment of Vero E6 and Calu-3 cells in triplicate. Cell Lysis Buffer (Cell Signaling Technology 9803S) treatment was used as the control for 100% cell death. The CellTox Green Cytotoxicity Assay (Promega) was performed according to the manufacturer’s instructions at 72 hours after treatment for all cell types in triplicate. Lysis Solution (Promega) treatment was used as the control for 100% cell death.

### Mutagenicity testing.

The standard AMES (*Salmonella typhimurium* reverse mutation assay, ref. 43) was performed by Cyprotex to evaluate the mutagenic potentials of sangivamycin. Approximately 10 million bacteria were exposed in triplicate to sangivamycin (6 concentrations, 1 mg/mL high test concentration), a negative control (vehicle), and a positive control for 90 minutes in medium containing a low concentration of histidine (sufficient for about 2 doublings). The cultures were then diluted into indicator medium lacking histidine and dispensed into 48 wells of 384-well plates (microplate format). Plates were incubated for 48 hours at 37°C. In the microsuspension assay, cells that undergo a mutagenic reversion grow, resulting in a color change that is scored as positive wells. Wells with bacterial growth were counted and compared with those with vehicle control. A dose-dependent increase in the number of colonies of at least 2-fold over baseline (mean + SD of the vehicle control) indicated a positive response. An unpaired, 1-sided Student’s *t* test was used to identify the conditions that were significantly different from those of the vehicle control.

### Potassium channel inhibition testing.

Ruling out inhibition of KCNH2 (hERG) by a novel compound is a mandatory step in establishing drug safety. The effect of sangivamycin on KCNH2-derived cardiac potassium channels was assessed using HEK293T cells and the QPatch HTX electrophysiology platform (Evotech), following the manufacturer’s instructions. Sangivamycin was tested at 6 concentrations (cumulative concentration response, high test concentration of 10 μM). The percentage change in potassium channel tail current was used to calculate channel inhibition.

### Metabolic stability testing.

To evaluate metabolic stability, sangivamycin was incubated by Cyprotex in duplicate with active and heat-inactivated human and CD-1 laboratory mouse liver microsomes (0.5 mg/mL) and cofactors (2.5 mM NADPH and 3.3 mM MgCl_2_) in 0.1 M phosphate buffer (pH 7.4) at 37°C. Aliquots were removed at 0, 15, 30, 60, 90, and 120 minutes, and the amount of drug remaining at each time point was determined by LC-MS/MS.

### CYP inhibition testing.

Sangivamycin was incubated by Cyprotex with a cocktail of model CYP substrates for CYP1A2, CYP2B6, CYP2C8, CYP2C9, CYP2C19, CYP2D6, CYP3A4, and CYP3A4, along with human liver microsomes (0.5 mg/mL) and cofactors (2.5 mM NADPH and 3.3 mM MgCl_2_), in 0.1 M phosphate buffer (pH 7.4) for 20 minutes at 37°C. Known CYP inhibitors were used in lieu of sangivamycin as positive controls: furafylin (1A2), thioTEPA (2B6), montelukast (2C8), sulfaphenazole (2C9), nootkatone (2C19), quinidine (2D6), and ketoconazole (3A4). Formation of substrate breakdown metabolites was measured by LC-MS/MS and compared with control incubation (incubation of substrates with microsomes and cofactors but in the absence of sangivamycin or in the presence of known inhibitors). CYP inhibition was measured as a decrease in the formation of expected metabolites in the presence of sangivamycin or control inhibitor (defined as a percentage of control).

### Turbidimetric aqueous solubility screening.

Aqueous solubility of sangivamycin was measured using a high-throughput turbidimetric assay performed by Cyprotex. Initially, sangivamycin stock DMSO solution was diluted in DMSO to produce a range of concentrations. These dilutions were added to PBS at pH 7.4 (high test concentration 500 μM) and incubated for 2 hours at 37°C. At the end of the incubation period, the absorbance at 620 nm was measured for each concentration for 2 replicates to determine turbidity from precipitate formation.

### Plasma protein binding assessment.

The plasma protein binding assay was performed by Cyprotex to assess sangivamycin plasma protein binding. Equilibrium dialysis was used to determine the extent of binding of a compound to plasma proteins. A semipermeable membrane separated a protein-containing compartment (PC) from a protein-free compartment (PF). The system was set to equilibrate at 37°C. The test compound present in each compartment was then quantified by LC-MS/MS. The extent of binding is reported as a fraction unbound (fu) value, which is calculated as fu = 1 – ([PC – PF]/PC).

### Mitochondrial dye cell imaging.

Huh-7 cells were untreated or treated with 100 nM sangivamycin for 5 days. Cells were then washed with PBS and incubated in serum-free media with fluorescent mitochondrial tracking dyes (2 μM 10 NAO and 0.4 μM MTG) for 30 minutes. Cells were washed with PBS and imaged at 10× original magnification using an IX81 inverted microscope (Olympus Life Science), a fluorescein isothiocyanate (FITC) filter cube, and an ORCA-05G digital camera with a charged-coupled device (CCD) (Hamamatsu Photonics).

### Acute mitochondrial or glycolytic stress assay.

Drug stocks were diluted in assay media–unbuffered DMEM (MilliporeSigma D5030-1L) with 1 mM D-glucose (MilliporeSigma G8270), 1 mM glutamine (MilliporeSigma G7513), 0.1 mM pyruvate (MilliporeSigma P2256), and 5 mM HEPES (MilliporeSigma H3375), pH 7.4 at 37°C. Huh-7 cells were washed once in warm filtered 1× PBS and then incubated in 0.1 mL assay media/well at 37°C for less than 1 hour. Drug injections made up in assay media were as follows: Port A (5x working stock): sangivamycin (0.05–5 μM final or DMSO); Port B (6x working stock): oligomycin (MilliporeSigma 75351) (1 μg/mL final); Port C (7x working stock): FCCP (MilliporeSigma C2920) (2 μM final); and Port D (8x working stock): rotenone (MilliporeSigma R8875)/antimycin A (MilliporeSigma A8674) (1 μM final) and 2-deoxyglucose (MilliporeSigma D8375) (15 mM final).

Seahorse assay cartridges were loaded in a Seahorse XF96e analyzer (Agilent; S7804-90001_REV A). Cells were counted using a Celligo Imaging Cytometer (Nexcelom Bioscience; 200-BFFL-S) using bright-field imaging and counting the inner 50% of each well area (to avoid misidentification of “high molded stops” as cells). XF96 cell culture microplates (Agilent; V3-PS TC-Treated) were loaded into the Seahorse XF96e analyzer, and following equilibration, the assay was run with the following cycles: baseline: mix 0.5 minutes, wait 0.5 minutes, measure 2 minutes (3 repeats); inject Port A: mix 0.5 minutes, wait 0.5 minutes, measure 2 minutes (10 repeats); inject Port B: mix 0.5 minutes, wait 0.5 minutes, measure 2 minutes (3 repeats); inject Port C: mix 0.5 minutes, wait 0.5 minutes, measure 2 minutes (3 repeats); and inject Port D: mix 0.5 minutes, wait 0.5 minutes, measure 2 minutes (3 repeats).

Data were collected using Seahorse Wave 2.0 software (Agilent) and processed using Microsoft Excel.

### Phospholipidosis imaging.

Phospholipidosis was assessed as previously described (32). Briefly, A549 cells were cultivated in Ham’s F12-K Medium (Thermo Fisher Scientific 21127-022) containing 10% FBS and seeded in a clear-bottom, 96-well plate at a density of 15,000 cells per well. The day after seeding, the cells were treated for 24 hours with a range of drugs at various doses (sangivamycin 5 to 0.04 μM, amiodarone 10 to 0.16 μM as a positive control, melperone 10 μM as a negative control) in the presence of 7.5 μM NBD-PE (Thermo Fisher Scientific N360). The final DMSO concentration was 0.1%. Cells were stained for 20 minutes at 37°C, 5% CO_2_, with media containing Hoechst 33342 (AnaSpec, Inc.) (10 μg/mL) and ethidium homodimer-2 (Thermo Fisher Scientific E3599) (2 μM), washed with Dulbecco’s PBS (Hyclone), and imaged by measuring NBD-PE fluorescence (excitation 463 nm/emission 536 nm) using a Synergy 4 plate reader with a GFP filter cube. The average fluorescence for DMSO-treated controls (*n* = 18) was set as the baseline fluorescence. A total of 10 μM of amiodarone was used as the positive control for phospholipidosis (*n* = 3), set as 100% fluorescence. Curves were generated comparing relative fluorescence for amiodarone and sangivamycin concentrations with Prism v9.2.0 (GraphPad Software). Cells were imaged at 10× original magnification using an IX81 inverted microscope; DAPI, FITC, and Texas Red filter cubes; and an ORCA-05G digital camera with a CCD. After imaging, cells were treated with CellTiter-Glo (Promega) for cell viability assessment.

### Statistics.

In Tables 2 and 3, 2-tailed Mann-Whitney *U* tests were used to compare independent groups. In Supplemental Table 1, a 2-tailed Welch’s *t* test was used to compare groups. In all figures, error bars represent standard deviations. All dose-response curves were fit to an asymmetric sigmoidal 5PL nonlinear regression curve with top and bottom constraints of 100 and 0, respectively, to calculate IC_50_, IC_90_, and CC_50_ values. Prism v9.2.0 software (GraphPad Software) was used for statistical analyses and nonlinear regression curve fitting. *P* values less than 0.05 were considered statistically significant.

### Study approval.

Cell lines were used, not human samples. Animal studies were done through a contract with NIAID Division of Microbiology and Infectious Diseases SRI Biosciences Study B119-18, whose ethics committee (SRI Biosciences Laboratory Animal Welfare, Menlo Park, California, USA) approved these studies.

## Author contributions

RPB, ENP, BPE, HCS, and JHK conceived and designed the experiments. RPB, ENP, BPE, YC, SY, COS, JL, HZ, GAK, and MJM performed experiments. RPB wrote the manuscript with significant interpretation and writing input from ENP, HCS, and JHK.

## Supplementary Material

Supplemental data

## Figures and Tables

**Table 1:**
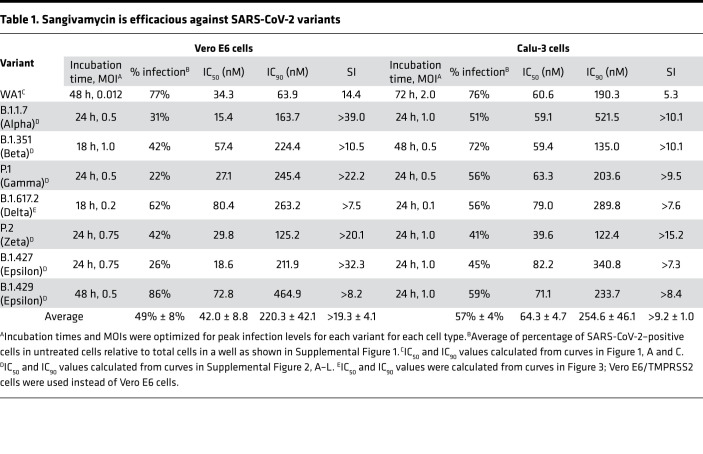
Sangivamycin is efficacious against SARS-CoV-2 variants

**Table 2:**
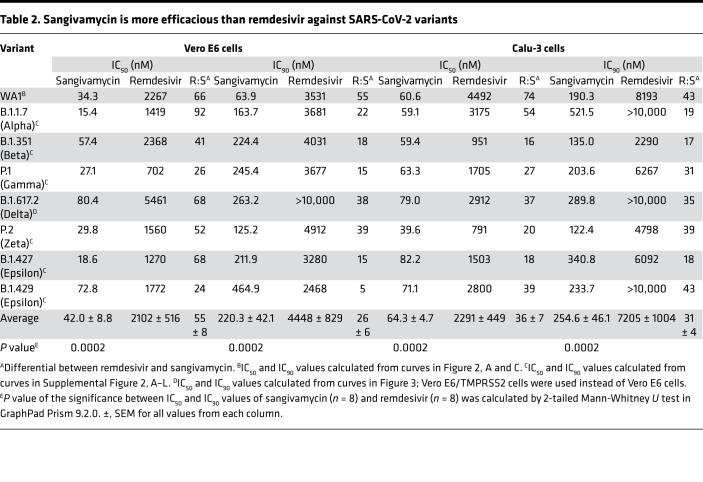
Sangivamycin is more efficacious than remdesivir against SARS-CoV-2 variants

**Table 3:**
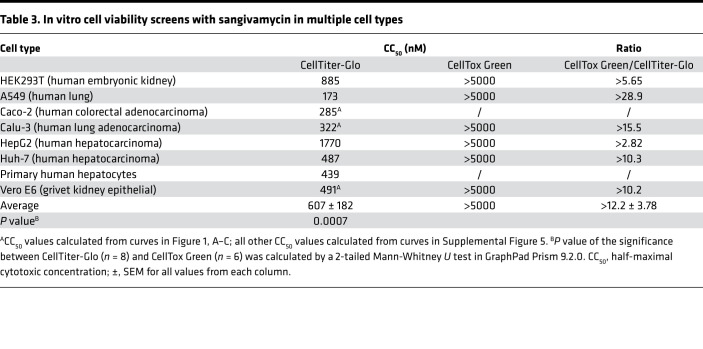
In vitro cell viability screens with sangivamycin in multiple cell types

**Table 4:**
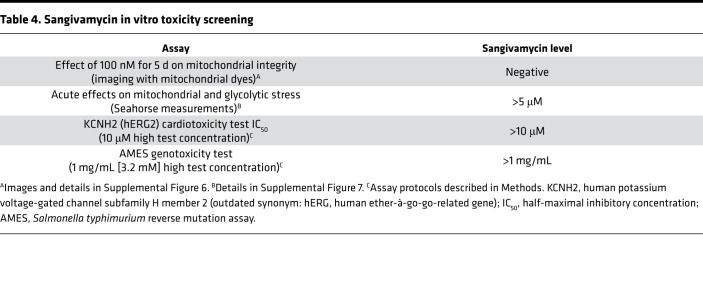
Sangivamycin in vitro toxicity screening

**Table 5:**
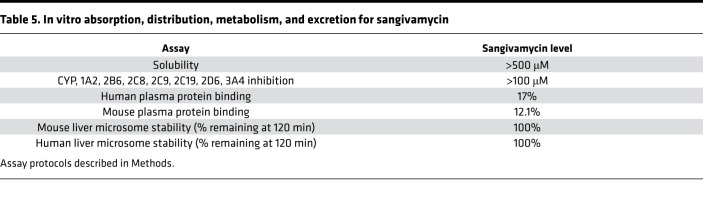
In vitro absorption, distribution, metabolism, and excretion for sangivamycin

## Version 1 11/22/2021

In-Press Preview

## Version 2 01/11/2022

Electronic publication
