# Supplementary material for: Sangivamycin is highly effective against SARS-CoV-2 in vitro and has favorable drug properties
Source: JCI Insight. 2022 Jan 11;7(1):e153165. doi: 10.1172/jci.insight.153165 (PMC8765048; doi:10.1172/jci.insight.153165)
Supplement: Supplemental data [file jciinsight-7-153165-s030.pdf]

Supplemental Figure 1

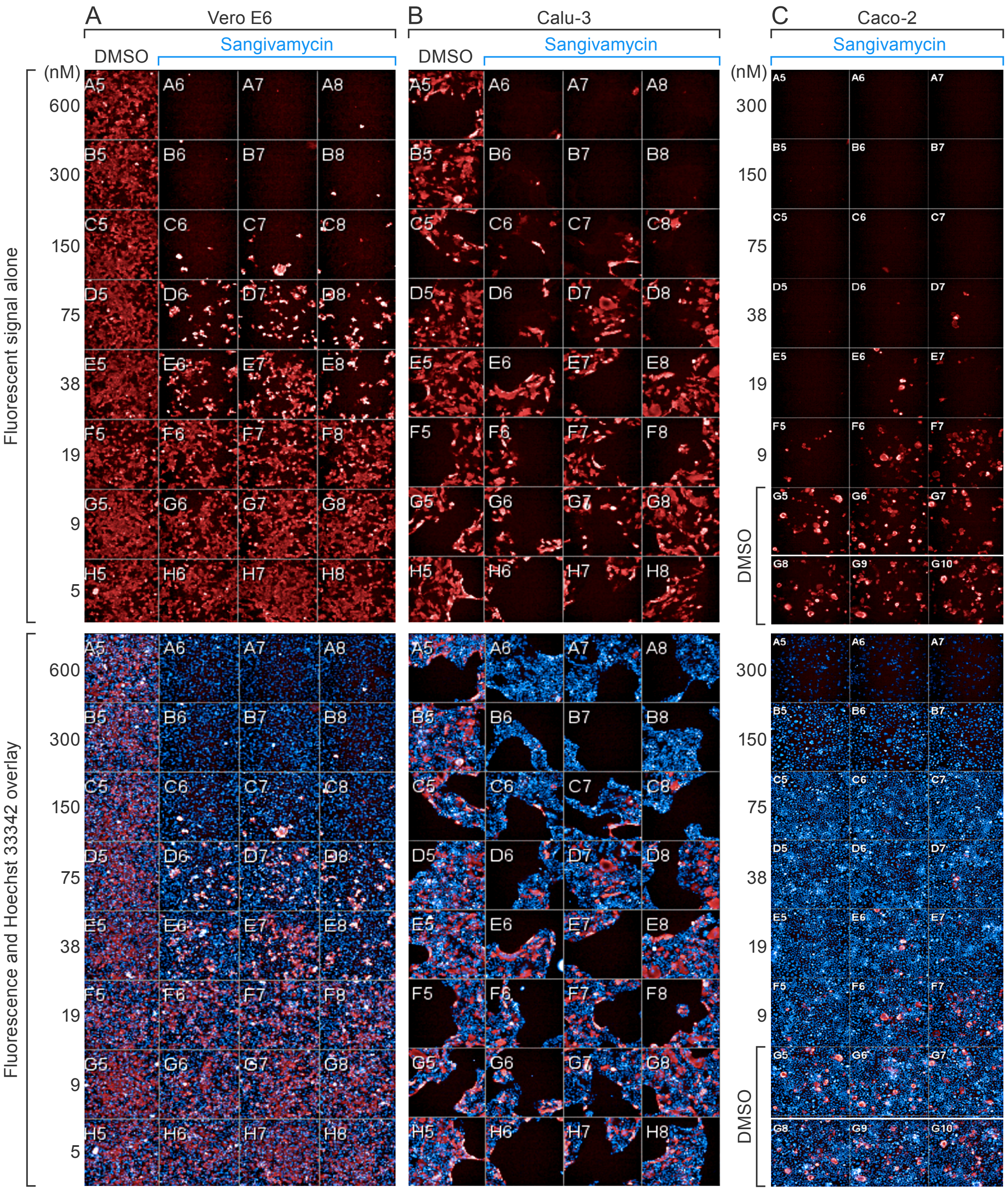

High content imaging to detect SARS-CoV-2 infectivity. Representative immunofluorescence images of fluorescently-labeled SARS-CoV Nucleoprotein (cross reactive with SARS-CoV-2) for Vero E6 (A), Calu-3 (B), and Caco-2 (C) cells. Top panel: Fluorescent signal; bottom panels: overlay of Fluorescence and Hoechst 33342 nuclear stains. (A and B) DMSO controls are in the left column and a triplicate dose series of sangivamycin (600 nM to 5 nM) in the right three columns. (C) A triplicate dose series of sangivamycin (300 nM to 9 nM) in the first six rows and the bottom two rows are DMSO controls. The percent infectivity was determined by calculating fluorescence-positive cells relative to Hoechst 33342-positive cells as a measure of total cell count in the well.

Supplemental Figure 2

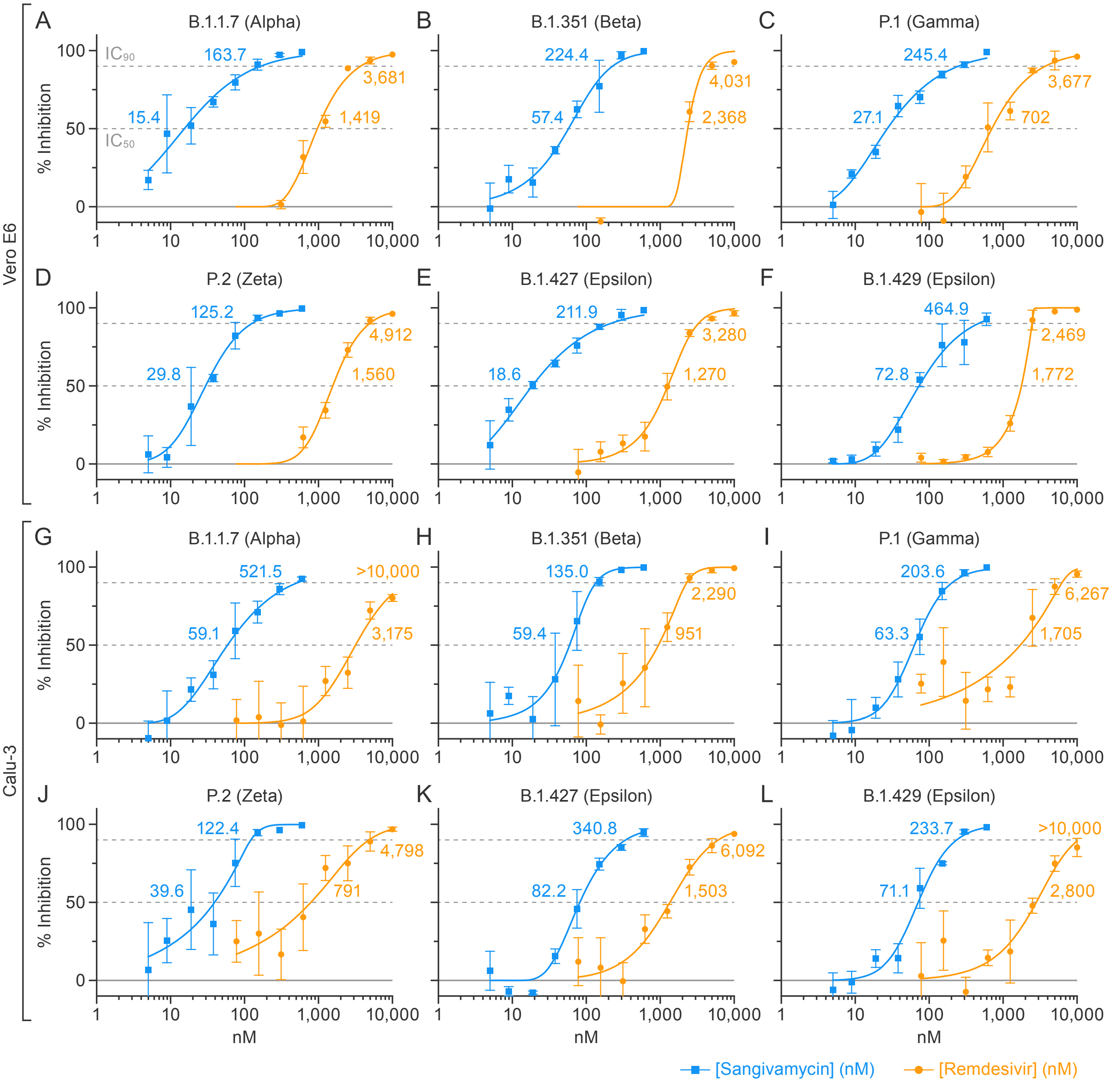

Sangivamycin outperforms remdesivir against SARS-CoV-2 variants. (A–L) Sangivamycin's antiviral activity compared to remdesivir using identical SARS-CoV-2 MOIs and sampling timepoints as detailed in Table 1 for each variant indicated above each graph. Results are reported as percent inhibition relative to untreated controls (blue values for sangivamycin, yellow values for remdesivir). Error bars represent standard deviations (SDs). IC50, half-maximal inhibitory concentration (lower dotted line); IC90, 90% inhibitory concentration (upper dotted line) are also listed in Table 2.

Supplemental Figure 3

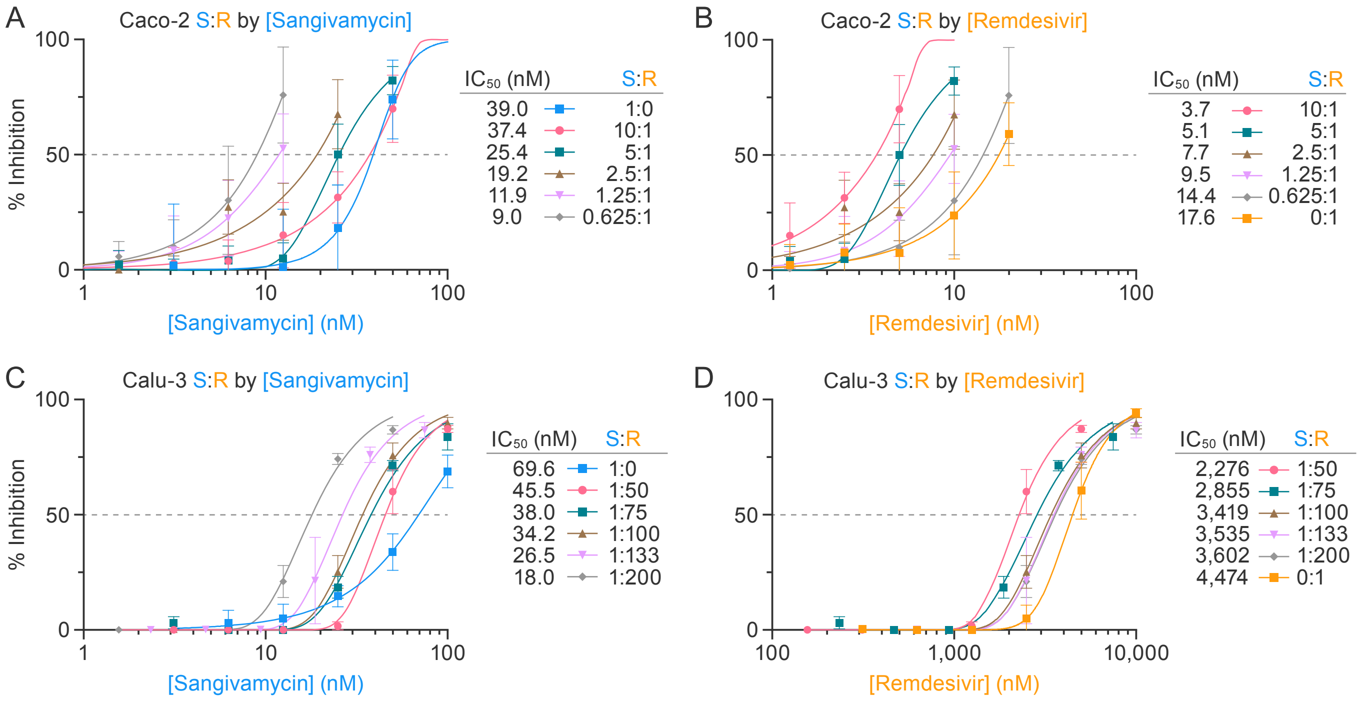

Combining sangivamycin and remdesivir results in an additive effect against SARS-CoV-2 in multiple cell types. Using results from Figures 2B and 2C, constant ratios of sangivamycin to remdesivir (S:R) were used to evaluate combination effect against SARS-CoV-2 infection in Caco-2 (A and B) and Calu-3 (C and D) cells and plotted relative to (A and C) sangivamycin concentration and (B and D) remdesivir concentration. Each dose combination was run in triplicate with error bars representing standard deviations (SDs). IC50, half-maximal inhibitory concentration.

Supplemental Figure 4

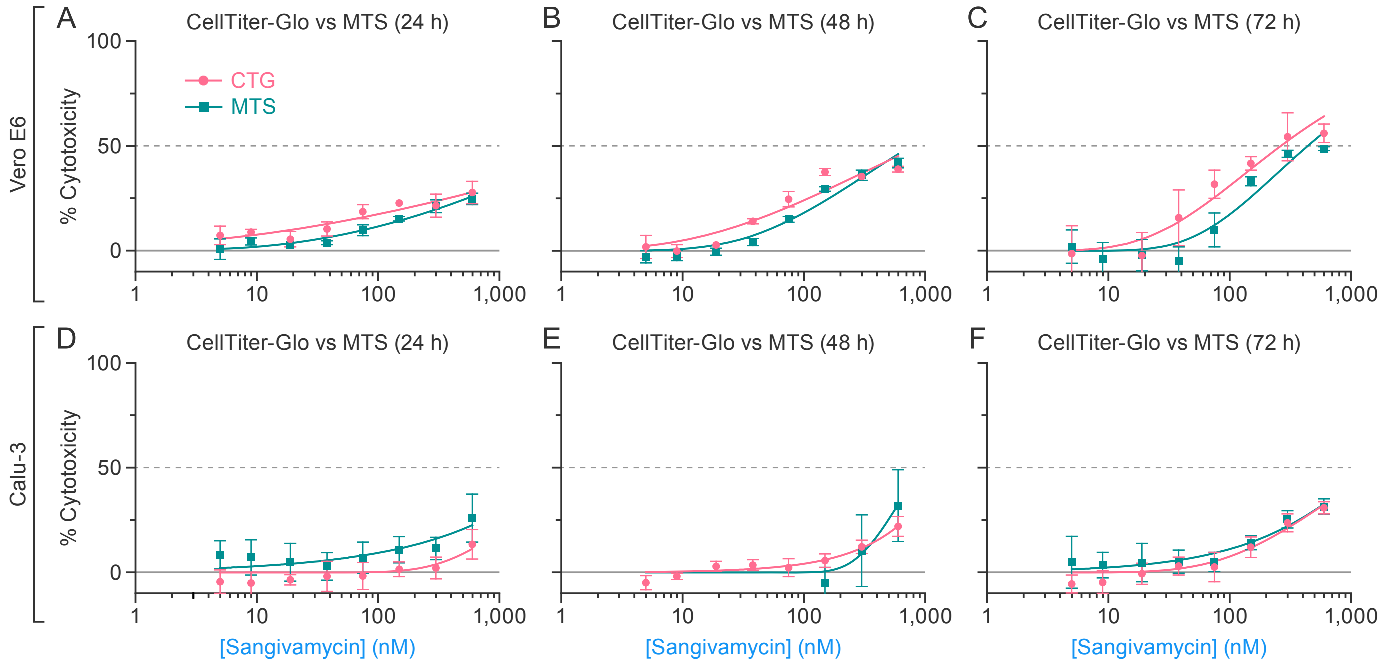

Cell viability assay comparisons at different timepoints in Vero E6 and Calu-3 cells. Cell viability dose responses curves were plotted using data from 24-h, 48-h, and 72-h measurements using MTS and CellTiter-Glo (CTG) assays in Vero E6 (A–C) and Calu-3 (D–F) cells treated with 600 nM to 5 nM sangivamycin run in triplicate with error bars representing standard deviations (SDs).

Supplemental Figure 5

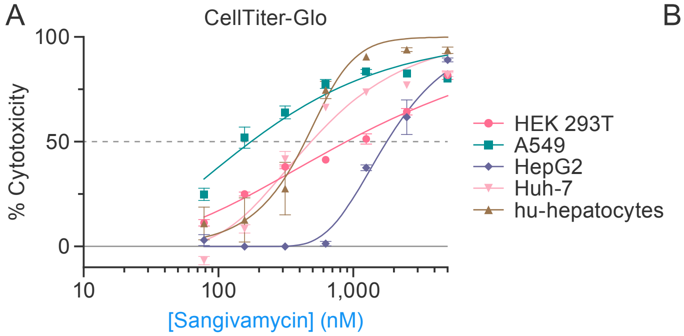

Cell viability assay comparisons in multiple cell types. Cell viability dose-responses curves were plotted using 72-h CellTiter-Glo (A) and CellTox Green (B) assay measurements of cells treated with 5,000 nM to 78 nM sangivamycin run in triplicate with error bars representing standard deviations (SDs). IC50 (half-maximal inhibitory concentrations) in Table 3 were generated from these curves.

Supplemental Figure 6

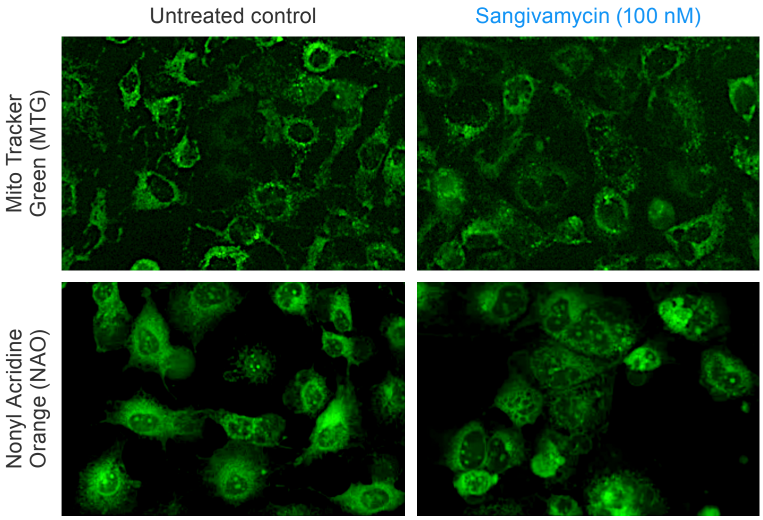

Mitochondrial structural integrity after five days of sangivamycin treatment. Fluorescent images of Huh-7 cells were untreated or treated with 100 nM sangivamycin for 5 d and exposed to fluorescent mitochondrial tracking dyes: 10 N-alkyl acridine orange (NAO) and MitoTracker Green (MTG).

Supplemental Figure 7

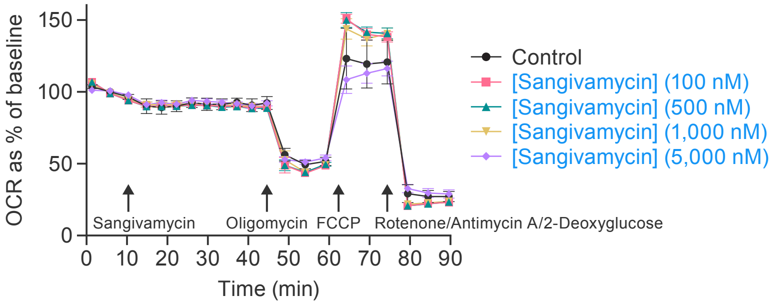

Sangivamycin does not induce acute mitochondrial or glycolytic stress. The graph shows the oxygen consumption rate (OCR) measured over time in minutes with the XF96 Seahorse instrument with varying sangivamycin concentrations and inhibitors relative to untreated control Huh-7 cells with error bars representing standard deviations (SDs) from an n=10 for sangivamycin treatments and n=6 for the control.

Supplemental Figure 8

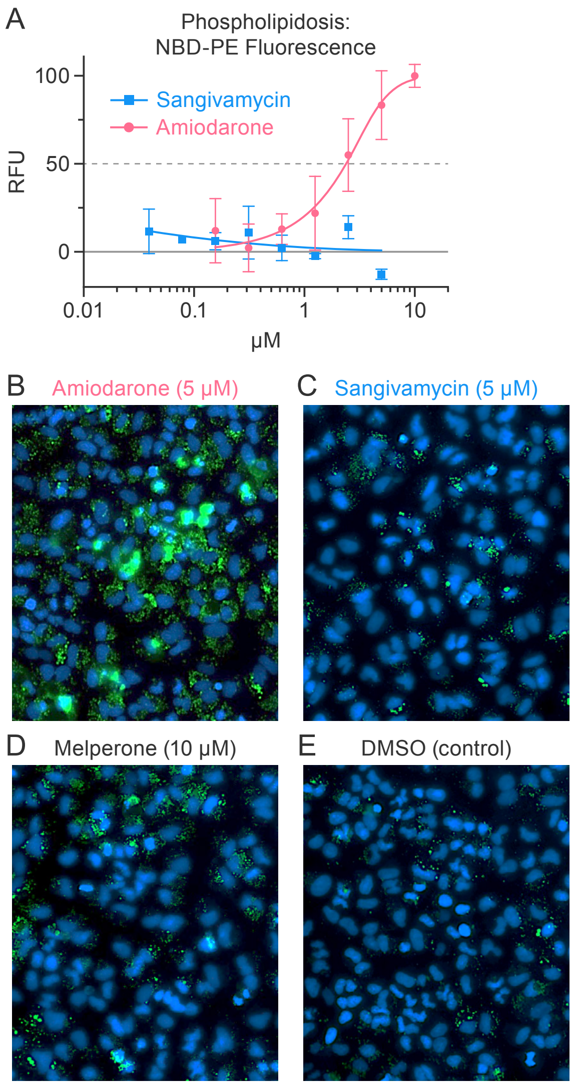

Sangivamycin does not induce phospholipididosis. (A) Dose-dependent increase in NBD-PE intracellular fluorescence in A549 cells using positive-control compound amiodarone over a dose range of 10 μM to 0.16 μM along with sangivamycin over a dose range of 5 μM to 0.04 μM (not resulting in an increase in fluorescence) run in triplicate with error bars representing standard deviations (SDs). (B–E) Fluorescence images of NBD-PE fluorescence with Hoechst 33342 overlayed for the positive control compound, 5 μM amiodarone (B), 5 μM sangivamycin (C), the negative control compound, 10 μM melperone (D), and the DMSO control (E).

Supplemental Figure 9

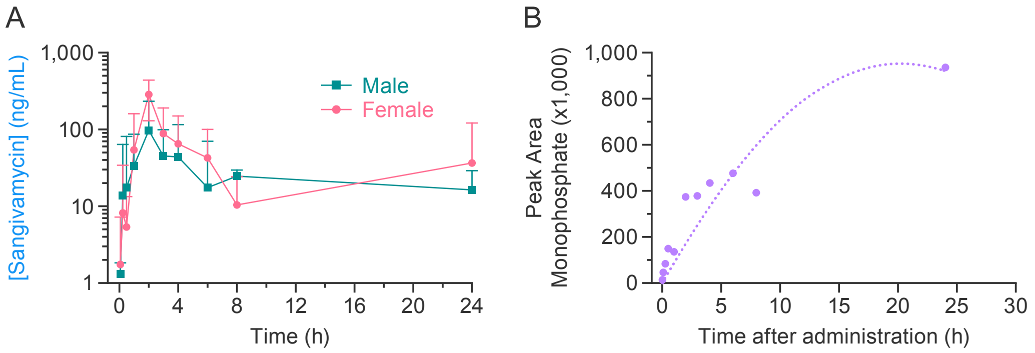

Sangivamycin 24-hour PK in laboratory mice. A) Pharmacokinetic study at SRI Biosciences. Three male and three female CD-1 laboratory mice received sangivamycin as a single i.p. dose at 60 mg/kg. Blood was collected at 5, 15, and 30 min and 1, 2, 3, 4, 6, 8, and 24 h after doing and processed to plasma. Clinical observations were performed immediately after doing and prior to the last blood collection. An LC-MS/MS bioanalytical method was developed and used to analyze plasma concentrations of sangivamycin in triplicate with error bars representing standard deviations (SDs). The lower limit of quantitation for the assay was 1 ng/mL. B) Monophosphate detection. Increase in peak area of sangivamycin monophosphate (SMP) with time after dosing. There is a peak in the SMP signal (m/z 388) at retention time ≈1.00–1.05 min (not present in control animal) that increased in intensity with time after dosing. The chromatographic peaks for SMP were integrated and then plotted as a function of time after dosing.

Supplemental Table 1

Sangivamycin inhibits SARS-CoV-2 replication in Vero E6 cells

**Drug**	**IC_{50} (nM) MOI 0.2**	**IC_{50} (nM) MOI 0.4**	**IC_{50} (nM) MOI 0.6**	**IC_{50} (nM) MOI 1.3**	**IC_{50} (nM) Average**
Sangivamycin	44 ± 6	89 ± 15	32 ± 4	43 ± 6	52 ± 13
GS-441524[A]	1,400 ± 60	1,440 ± 110	/	/	1,420 ± 20
Differential GS-441524/sangivamycin	32	16	/	/	27
P-value*	0.0005				

MOI = multiplicity of infection (SARS-CoV-2)
[A]GS-441524 is the parent nucleoside of remdesivir.
± was calculated as the standard error of the mean (SEM) for duplicate plates for each MOI.
*P-value determined by a two-tailed Welch's t test in GraphPad Prism 9.2.0

Supplemental Table 2

SARS-CoV-2 Variant Information

Lineage	WHO Name	IRF Lot Number	Source Isolate	Prod. Cell Line	GenBank #
WA1		IRF0394	USA_WA1/2020	Vero CCL-81	MW161259
WA1		IRF0399	USA_WA1/2020	Vero CCL-81	MT952134
B.1.117	Alpha	IRF0427	CA-0271 B1	Vero CCL-81	MW981411
B.1.351	Beta	IRF0434	hCoV-19/South Africa/KRISP-K005325/2020	Vero E6	MZ376663
P.1	Gamma	IRF0451	hCoV-19/Japan/TY7-503/2021	CALU-3	OK091603
B.1.617.2	Delta	IRF0455	SARS-CoV-2, hCoV-19/USA/PHC658/2021	CALU-3	Pending
P.2	Zeta	IRF0445	hCoV-19/USA/CA-VRLC012/2021 Brazil P.2	Vero CCL-81	MZ375761
B.1.429	Epsilon	IRF0438	hCoV-19/USA/CA-CDC-50070/2020	Vero CCL-81	MZ376658
B.1.427	Epsilon	IRF0442	hCoV-19/USA/CA-CDC-48018/2020	CCL-81	MZ376661
